# Ubiquitin-Specific Peptidase 22, a Histone Deubiquitinating Enzyme, Is a Novel Poor Prognostic Factor for Salivary Adenoid Cystic Carcinoma

**DOI:** 10.1371/journal.pone.0087148

**Published:** 2014-01-23

**Authors:** Wei Dai, Yuan Yao, Qing Zhou, Chang-fu Sun

**Affiliations:** 1 Department of Oromaxillofacial-Head and Neck Surgery, Department of Oral and Maxillofacial Surgery, School of Stomatology, China Medical University, Heping District, Shenyang, Liaoning,China; 2 Department of Gastroenterology, the People’s Hospital of Liaoning Province, Shenyang, Liaoning, China; Ospedale Pediatrico Bambino Gesu', Italy

## Abstract

Salivary Adenoid Cystic Carcinoma (SACC) is characterized by a high rate of local recurrence and infiltration, strong invasion to peripheral nerves or late distant metastasis. Our aim was to investigate the expression of Ubiquitin-specific protease 22 (USP22) in SACC patients and its possible relationship to the outcome of the disease. A total of 135 SACC tissues and adjacent non-cancerous tissues which were diagnosed between 2002 and 2007 were enrolled in this study. Immunohistochemistry was used to compare the expression pattern of USP22 in SACC and adjacent non-cancerous groups, and the prognostic significance was assessed by Kaplan-Meier analysis and Cox proportional hazards regression in SACC patients. The rate of high expression of USP22 was significantly higher in SACC group than that in adjacent non-cancerous group. High expression of USP22 was significantly correlated with histological subtype, lymph node metastasis, grade, Ki-67 and SOX2 expression. Furthermore, USP22 acts as an oncogene by regulation the BMI-1 pathway and c-Myc pathway. SACC patients with high USP22 expression showed the poorer overall survival (OS) and disease-free survival (DFS) than those patients with low USP22 expression. In multivariate analysis, only lymph node metastasis and USP22 expression were the independent prognostic factors for OS and DFS in SACC. Our study provides evidence that USP22 expression is an independent prognostic factor for SACC patients.

## Introduction

Salivary Adenoid Cystic Carcinoma (SACC) is characterized by a high rate of local recurrence and infiltration, strong invasion to peripheral nerves or late distant metastasis [1], [2] and is one of the most common malignant tumors of salivary gland, which accounts for 24% of salivary gland neoplasms [3]. Due to high rates of recurrence and metastases, patients with SACC have a poorer disease specific survival [4], [5]. The elevated expression of HIF-2α, TWIST2, and SIP1 has been reported to contribute to invasion and metastasis of SACC [6]. However, predictive and prognostic factors of SACC phenotype are poorly understood. Therefore, there is an urgent need to identify new prognostic biomarkers that can be used to predict a therapeutic response and clinical outcomes in SACC patients to rationalize treatment decisions.

Recently, increasing evidence shows that Ubiquitin-specific protease 22 (USP22) have been recognized as a novel histone deubiquitinating enzyme, which is involved in tumor development and progression [7]. To date, several studies have reported USP22 was predicted as a poor prognostic factor in patients with non-small cell lung cancer, salivary duct carcinoma, papillary thyroid carcinoma and oral squamous cell carcinoma [7], [8], [9], [10]. In addition, Sussman RT, *et al.* have reported that USP22 promotes embryonic stem cell differentiation through transcriptional repression of Sex determining region Y-box 2 (Sox2) [11]. USP22 regulates cell-cycle progression via both INK4a/ARF pathway and Akt signaling pathway in human colorectal cancer [12]. Thus, identification of the relationship between USP22 expression and clinicopathological features and prognosis of SACC patients is critical, which will help us to further understand the pathogenesis of SACC therapeutics.

In this study, we determined the expression of USP22 in SACC tissues, and found that USP22 expression was upregulated in SACC tissues and correlated with histological subtype, lymph node metastasis, grade, Ki-67, and SOX2 expression in SACC patients. Moreover, USP22 acts as an oncogene by regulation the BMI-1 pathway and c-Myc pathway in ACC-83 cancer cell lines. Our results strongly indicate that overexpression of USP22 was a poor prognostic factor for SACC patients, which may lead to SACC invasion, metastasis and cell proliferation.

## Materials and Methods

### Ethics Statement

The formalin-fixed paraffin embedded specimens used to immunohistochemistry were collected from 135 SACC patients undergoing surgery between 2002 and 2007. Data were retrieved from patients’ operative and pathological reports, and follow-up data were obtained by the clinical database. No patient received preoperative chemotherapy or radiotherapy. Clinicopathological features of the patients were collected by the retrospective review of medical archives. The use of tissues for this study has been approved by the Human Research Ethical Committee of the affiliated Stomatological Hospital of China Medical University. At the time of initial diagnosis, all patients had provided consent in the sense that their tumor samples could be used for investigational purposes. Written informed consents were received from all participants involved in the study.

### Immunohistochemical Staining

Paraffin sections were cut at 4 µm thickness, mounted on polylysine coated slides and incubated overnight at 55 °C. Sections were deparaffinized in xylene and rehydrated with graded alcohol. Antigen retrieval was performed using citrate buffer (pH 6.0) and sections were held in Tris buffered saline (TBS). Endogenous peroxidase activity was blocked by incubation in 3% hydrogen peroxide. The slides were then incubated with USP22 rabbit polyclonal antibody (Abcam, OR, USA) at 1∶100 dilution and Ki-67 mouse monoclonal antibody (Maixin, Fuzhou, China). Staining for both antibodies was performed at 4°C overnight. The slides were incubated with horseradish peroxidase-conjugated goat anti-rabbit IgG, and the color was developed with the DAB Horseradish Peroxidase Color Development Kit (Maixin Co., Fuzhou, China) [13].

### Evaluation of Immunostaining

All the immunoreactions were separately evaluated for positive DAB staining by two independent pathologists. Five views were examined per slide, and 100 cells were observed per view at ×400 magnification. Cytoplasmic and nuclear immunostaining in tumor cells was considered positive staining. The percentage of positively stained cells exhibiting USP22 and SOX2 expression was scored as ‘0’ (0%), ‘1’ (1%–5%), ‘2’ (5%–25%), ‘3’ (25%–50%) and ‘4’ (50%–100%). Intensity was scored as 0: negative, 1: weak, 2: moderate and 3: strong. The staining intensity score plus the percentage of positive staining was used to define the USP22 and SOX2 expression levels: 0–2, low expression and 3–7, high expression, which classified SACC patients into two groups. Then, samples with Ki-67 nuclear staining equal or above 30% were considered having a high proliferative index, whereas nuclear positivity below 30% was considered a low proliferative index [14].

### Western blot

Cancer tissues were lysed with RIPA buffer (Sigma-Aldrich, Germany) and 50 µg of total protein was separated through electrophoresis on a SDS-PAGE gel and transferred to PVDF membranes (GE Healthcare, Barrington, IL, USA). The membrane was blocked at RT for 1 h in Tris-buffered saline (TBS) containing 0.1% Tween-20 (TBST) and 5% fat-free powdered milk, and incubated overnight with primary antibodies (USP22 antibody and GAPDH antibody) at 4°C. Then incubated with the secondary antibody for 1 h, and washed three times for 10 min in TBST prior to chemiluminescence detection (GE Healthcare, Barrington, IL, USA).

### Cell transfection, RNA extraction and reverse transcription–real-time PCR

Cells were grown in a 6-well culture plate to 70–80% confluence and then transfected with Control vector or Flag-USP22 vector using lipofectamine™ 2000 (Invitrogen, Carlsbad, CA, USA) according to the manufacturer’s instructions. Total RNA from cells was extracted using Trizol (Invitrogen, Carlsbad, CA, USA) and reverse transcribed to cDNA using Quantitect Reverse Transcription Kit (TaKaRa, Shiga, Japan). The SYBR green dye (Takara, Shiga, Japan) was used for the amplification of cDNA. The mRNA levels of USP22, BMI-1, p14ARF, c-Myc and cyclin D2, as well as that of the internal standard β-actin, were measured by real-time quantitative PCR in triplicate using an Mx3000P™ Real-Time PCR System by Agilent (Stratagene, La Jolla, CA, USA). The specific primers used for these genes are listed in Table 1.

**Table 1:** Sequence of Primers.

Gene	Primers (F: Forward; R: Reverse)	Amplicon size (bp)
USP22	F: 5′-GCTGCATTCCTGCCTCTA-3′	176
	R: 5′-CCTCCTTGGCGATTATTT-3′	
BMI-1	F:5′- CTCTTCTTGTTTGCCTAG-3′	160
	R:5′- TGATGACCCATTTACTGA-3′	
p14ARF	F:5′- TTCCTGGACACGCTGGTGGT-3′	169
	R:5′- CTATGCGGGCATGGTTACTGC-3′	
c-Myc	F:5′- CTGCGACGAGGAGGAGAA-3′	178
	R:5’- CCGAAGGGAGAAGGGTGT-3′	
cyclin D2	F:5′- GGAACAGAAGTGCGAAGAA-3′	190
	R:5′- GATGGAGTTGTCGGTGTAAAT-3′	
β-actin	F:5′-GGAAATCGTGCGTGACATT-3′	113
	R:5′-CAGGCAGCTCGTAGCTCTT-3′	

### Statistical analysis

SPSS version 13.0 software (SPSS, Chicago, IL, USA) for Windows was used for all statistical analyses. A χ2 test was used to examine possible correlations between USP22 expression and clinicopathological characteristics for the results of immunohistochemistry. One-way analysis was used to analyze the correlation between USP22 expression and SOX2 expression. OS was calculated from SACC diagnosis to the date of death for any cause, and DFS was defined as the time from SACC diagnosis to any event related to oral cancer. The Kaplan-Meier curves were plotted to calculate 5-year survival curves, and log-rank test was used to estimate the differences. OS and DFS curves were constructed to demonstrate the survival differences between the USP22-positive and USP22-negative patients. The impact of USP22 expression on patient OS and on DFS was assessed with the hazards regression (HR), calculated by both univariate and multivariate Cox proportional HR models. Differences were considered statistically significant when *p* values were <0.05.

## Results

### The Expression Profiles of USP22 and Ki-67 in SACC Tissues

**Figure 1:**
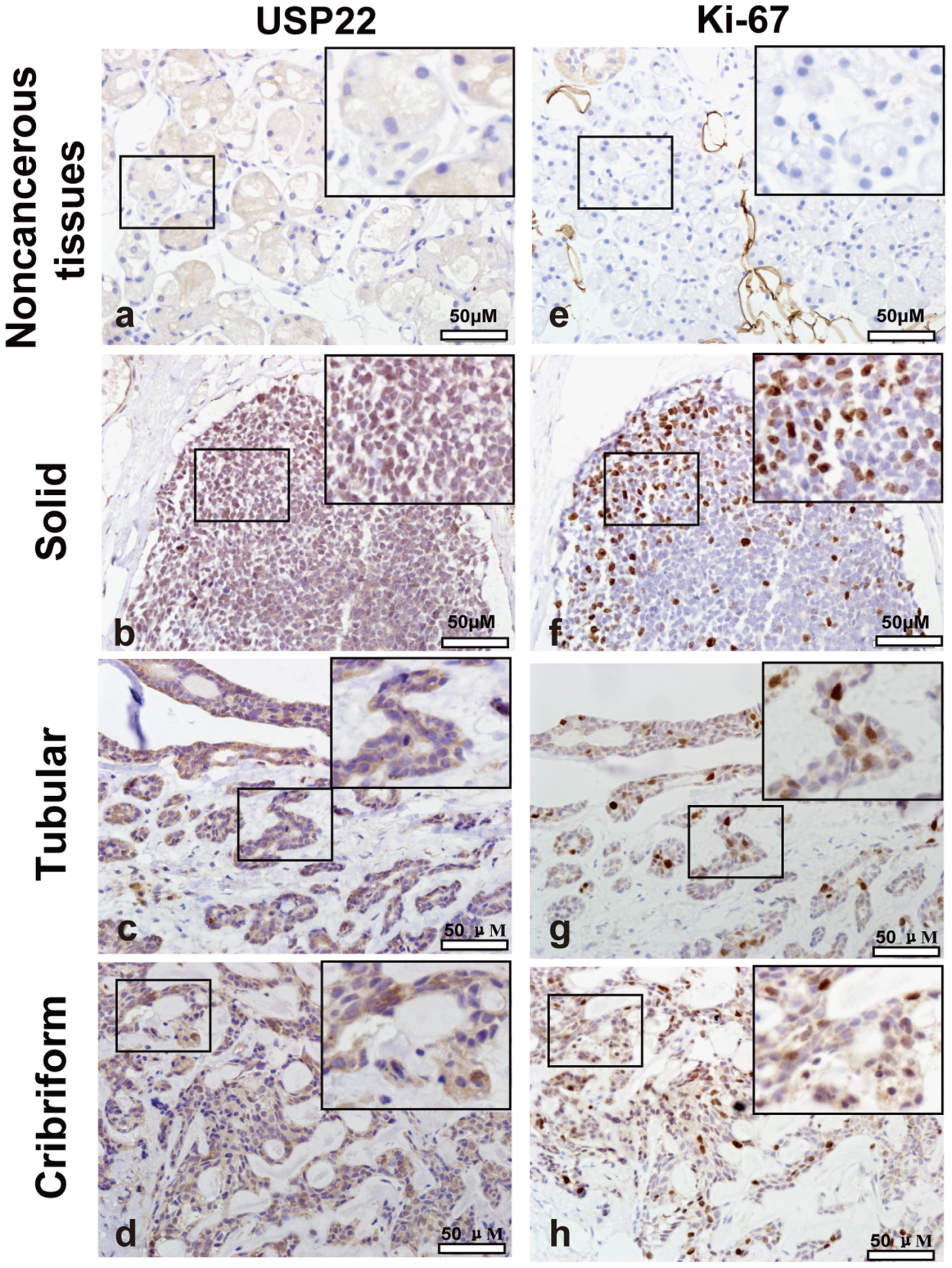
Immunohistochemistry for USP22 and Ki-67 in the SACC and adjacent non-cancerous tissues. (a) USP22 immunostaining in adjacent non-cancerous tissue. (b-d) USP22 immunostaining in SACC tissues. Positive USP22 staining in SACC tissues appeared as brown particles which were mainly localized within the nuclei and cytoplasm of epithelial cells of glands, whereas in the adjacent non-cancerous tissues, USP22 immunoreactivity was observed in the cytoplasm. (e) Ki-67 immunostaining in adjacent non-cancerous tissue. (f-h) Ki-67 immunostaining in SACC tissues. Positive Ki-67 staining in SACC tissues appeared as brown particles which were localized within the nuclei. Original magnification: all×400. Scale bar, 50 µm.

The clinical characteristics of SACC patients were shown in Table 2. The expression of USP22 and Ki-67 in SACC samples and normal salivary gland adjacent to tumor were analyzed by immunohistochemistry. As shown in Figure 1, USP22 was mainly expressed in the nucleus of the cancer cells, and occasionally in the cytoplasm. Further analysis revealed that 76.3% (103/135) of SACC specimens showed high expression of USP22, in contrast to low expression in SACC tissues. In all 135 SACC patients, high expression of Ki-67 was observed in 71.1% (96/135) of SACC patients.

**Table 2:** Correlation between USP22 expression and clinicopathological parameters in SACC patients.

Parameters	Low USP22 expression (n = 32)	High USP22 expression (n = 103)	χ^2^ value	*p-*value
Age(years)				
≤ 50	11 (34.4%)	31 (30.1%)	0.208	0.648
> 50	21 (65.6%)	72 (69.9%)		
Gender				
Male	18 (56.2%)	67 (65.0%)	0.811	0.368
Female	14 (43.8%)	36 (35.0%)		
Histological subtype				
Solid	2 (6.2%)	23 (22.3%)	4.184	0.041
Cribriform/Tubular	30 (93.8%)	80 (77.7%)		
LN metastasis				
Negative	26 (81.2%)	48 (46.6%)	11.834	0.001
Positive	6 (18.8%)	55 (53.4%)		
Tumor size				
≤ 2 cm	17 (53.1%)	65 (63.1%)	1.020	0.312
> 2 cm	15 (46.9%)	38 (36.9%)		
Grade				
I, II	25 (78.1%)	55 (53.4%)	6.183	0.013
III	7 (21.9%)	48 (46.6%)		
Nerve invasion				
Yes	19 (59.4%)	56 (54.4%)	0.248	0.619
No	13 (40.6%)	47 (45.6%)		
Ki-67				
Low expression	17 (53.1%)	22 (21.4%)	11.992	0.001
High expression	15 (46.9%)	81 (78.6%)		

### Correlation between USP22 expression and clinicopathological factors of SACC patients

By statistical analysis (Table 2), it was shown that the level of USP22 expression was significantly correlated with histological subtype (*P*  =  0.041), lymph node metastasis (*P*  =  0.001), grade (*P*  =  0.013), as well as the expression of Ki-67 (*P*  =  0.001), suggesting that high USP22 expression group showed a higher incidence of Solid subtype (22.3%), lymph node metastasis (53.4%), more advanced clinical stage (46.6%) and higher Ki-67 expression (78.6%) than the low USP22 expression group (6.2%, 18.8%, 21.9% and 46.9%, respectively).However, there were no significant differences between USP22 expression and other clinicopathological facors, including age (*P*  =  0.648), gender (*P*  =  0.368), tumor size (*P*  =  0.312) and nerve invasion (*P*  =  0.619).

### Survival analysis correlation of USP22 Expression in SACC with clinicopathological characteristics

**Figure 2:**
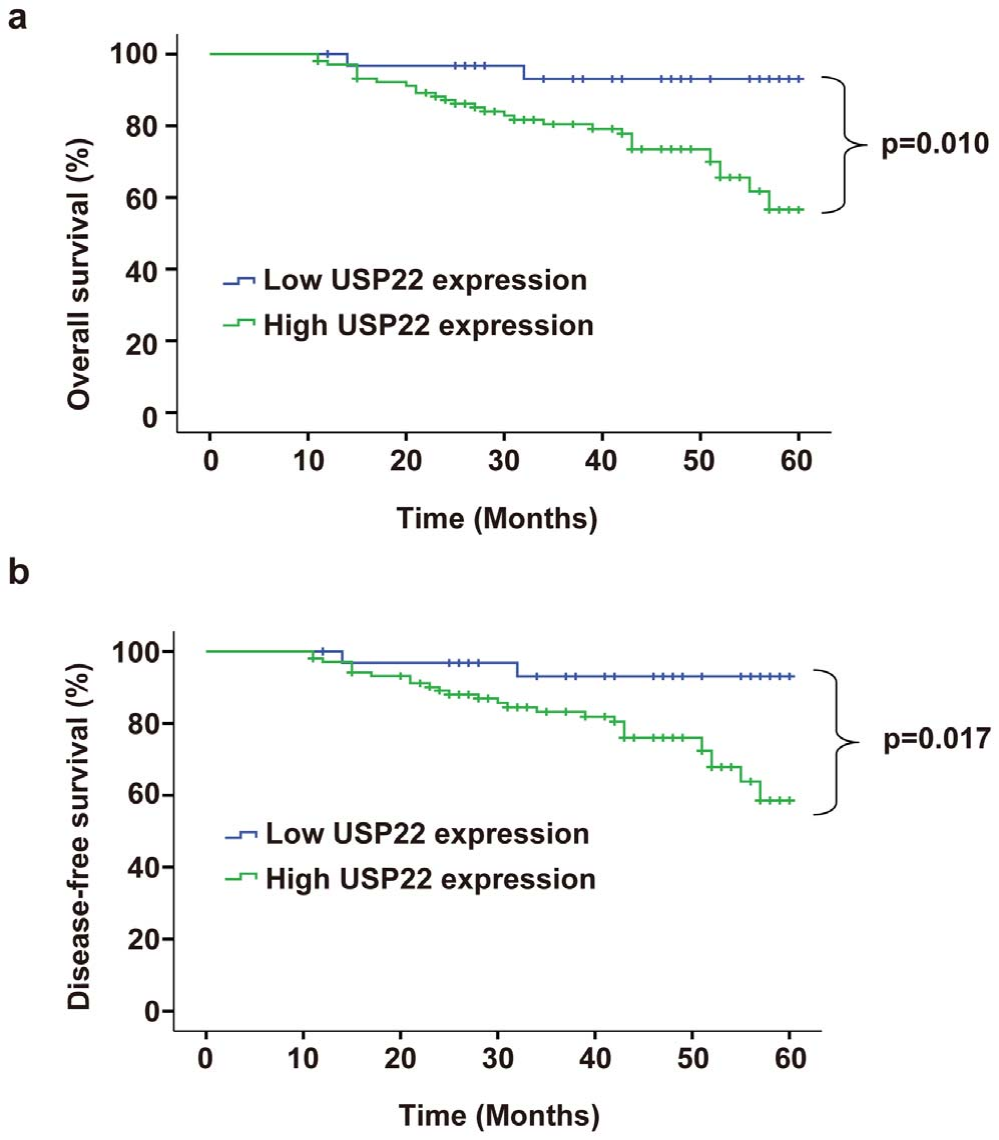
Kaplan-Meier survival curves for overall survival (OS) and Disease-free survival (DFS) OF SACC patients stratified by USP22 expression. (a) OS curves of SACC patients according to USP22 immunostaining; (b) DFS curves of SACC patients according to USP22 immunostaining. *p*-Values were obtained by log-rank test.

To elucidate the prognostic role of USP22 expression in SACC patients, we examined the relationship between USP22 expression and patient outcome with long-term follow-up. Overall survival (OS) and Disease-free survival (DFS) rates were estimated by Kaplan–Meier survival curves. The OS and DFS of SACC patients were shown in Figure 2. Patients with high expression of USP22 had shorter OS (P  =  0.010) and DFS (P  =  0.017) than those with low USP22 expression. Univariate and multivariate analyses were carried out using Cox proportional hazard model to evaluate the impact of USP22 expression and clinicalpathological factors on the prognosis of SACC patients. As shown in Table 3, univariate Cox regression analysis suggested that histological subtype (*P*  =  0.039 for OS; *P*  =  0.041 for DFS), lymph node metastasis (*P  = * 0.031 for OS; *P*  =  0.037 for DFS), grade (*P*  =  0.042 for OS; *P*  =  0.048 for DFS), Ki-67 expression (*P*<0.001 for OS and DFS), as well as USP22 expression (*P*  =  0.025 for OS; *P*  =  0.029 for DFS) were significantly associated with poor OS and DFS. Multivariate analyses showed lymph node metastasis (*P  = *0.041 for OS; *P*  =  0.045 for DFS), Ki-67 expression (*P*  =  0.005 for OS; *P*  =  0.008 for DFS), as well as USP22 expression (*P*  =  0.043 for OS; *P*  =  0.047 for DFS) were associated with poor OS and DFS.

**Table 3:** Univariate and multivariate Cox Proportional Hazards Model for OS and DFS in SACC patients.

Variables	Univariate			Multivariate		
	HR	95% CI	*p-*value	HR	95% CI	*p-*value
OS						
Age (≤50 *vs.* >50 years)	1.232	0.573–2.061	0.528			
Gender(Male vs. Female)	1.429	0.751–2.009	0.513			
Histological subtype (Solid vs. Cribriform/ Tubular)	3.267	0.521–3.908	0.039	2.624	1.527–3.418	0.058
Lymph node metastasis (Negative vs. Positive)	3.573	1.647–4.627	0.031	3.017	2.394–4.383	0.041
Tumor size (≤2 cm vs. >2 cm)	1.079	0.824–2.821	0.795			
Grade (I, II vs. III)	2.693	1.003–4.198	0.042	1.759	1.263–5.034	0.257
Nerve invasion (no vs. yes)	2.387	0.967–3.582	0.157			
Ki-67 expression (Low vs. High)	5.162	2.946–7.119	<0.001	4.201	2.143–8.771	0.005
USP22 expression (Low vs. High)	3.941	1.062–5.877	0.025	3.251	2.517–4.591	0.043
DFS						
Age (≤50 *vs.* >50 years)	1.135	0.614–2.007	0.617			
Gender(Male vs. Female)	1.294	0.819–2.267	0.598			
Histological subtype (Solid vs. Cribriform/ Tubular)	2.964	0.501–3.820	0.041	2.031	0.421–3.015	0.128
Lymph node metastasis (Negative vs. Positive)	3.187	1.394–4.296	0.037	2.716	0.954–3.771	0.045
Tumor size (≤2 cm vs. >2 cm)	1.022	0.755–2.691	0.836			
Grade (I, II vs. III)	2.508	0.991–3.315	0.048	1.359	0.408–2.598	0.504
Nerve invasion (no vs. yes)	2.154	0.864–3.382	0.220			
Ki-67 expression (Low vs. High)	5.031	2.772–7.068	<0.001	3.907	1.952–7.985	0.008
USP22 expression (Low vs. High)	3.624	1.059–5.718	0.029	3.001	1.726–4.915	0.047

OS, overall survival; DFS, Disease-free survival; HR, Hazard ratio; CI, confidence interval.

### Expression of USP22 in clinical SACC cancer samples

**Figure 3:**
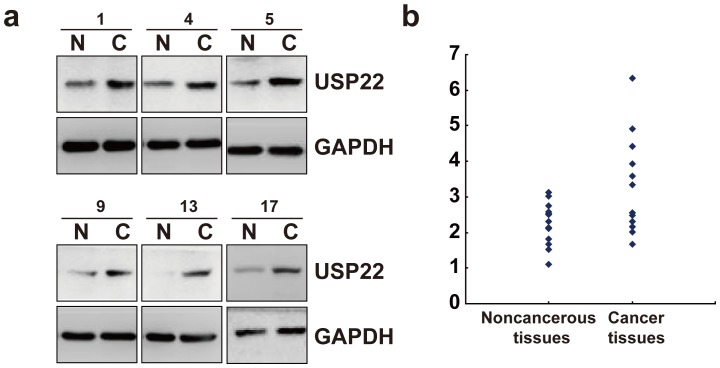
The expression of USP22 in SACC. (a) Evaluation of the expression of the indicated proteins in clinical tissues using western blot. Lysates of 20 cancer tissues (C) and matched adjacent noncancerous tissues (N) pairs were analyzed using western blot. The representative six pairs are shown. (b) Representative USP22 mRNA (normalized by GAPDH) is blotted on the scatter diagram. GAPDH, glyceraldehyde-3-phosphate dehydrogenase.

In order to further support the expression of USP22 in SACC, we further analyzed the expression of USP22 in freshly frozen SACC cancer tissues and matched adjacent noncancerous tissues from 20 SACC tumor patients using western blot analysis. The results showed that USP22 was clearly increased in 6 out of 20 analyzed cancer tissues (Figure 3a). A variation was observed among cases, whereas most of SACC tissues demonstrated increased expression of USP22 when compared with matched adjacent noncancerous tissues (Figure 3b).

### Correlation of USP22 with SOX2 in clinical SACC samples

**Figure 4:**
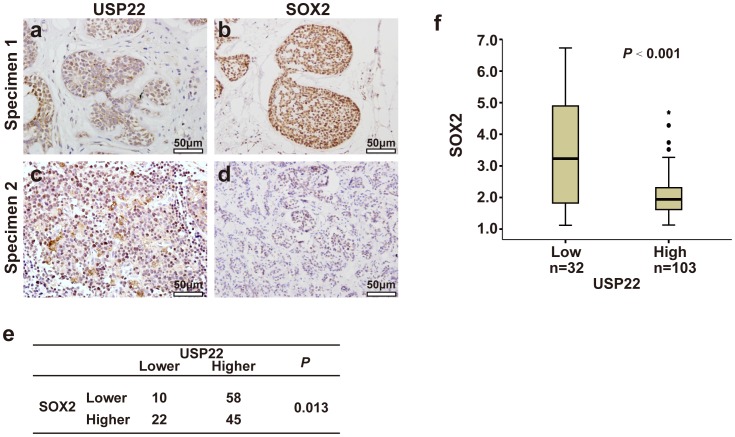
Correlation of USP22 and SOX2 in SACC. (a-d) SACC from serial sections of two patients (specimen 1 and 2) were subjected to immunohistochemical staining of USP22 (a and c) and SOX2 (b and d). (a) intermediate expression of USP22, (c) strong expression of USP22, (b) strong expression of SOX2, (d) intermediate expression of SOX2. Original magnification, ×400. Intensity values are expressed as Evaluation of Immunostaining. Scale bars  =  50 µm. (e) Higher expression of USP22 in SACC was significantly correlated with lower expression of SOX2. The *P*-value was generated using the χ^2^-test. (f) Box plot of USP22 and SOX2 expressions. The USP22 and SOX2 expression scores are shown as box plots, with the horizontal lines representing the median; the bottom and top of the boxes representing the 25th and 75th percentiles, respectively; and the vertical bars representing the range of data. In addition, extreme cases are marked with a dot. Data were analyzed by one-way analysis of variance (ANOVA) test.

In order to further support the roles of USP22 in SACC as well as to substantiate the functional link between USP22 and SOX2, we performed immunochemical staining of USP22 and SOX2 in a larger sample of clinical sections. USP22 expression correlated negatively with SOX2 expression (*P*  =  0.013) (Figures 3a-e). Furthermore, we divided tumor samples into two groups on the basis of USP22 amounts and studied the differences of SOX2 expression. The ANVOA analysis showed the expression scores of SOX2 in tumors were negatively regulated by USP22 expression (P<0.001; Figure 4f).

### USP22 acts as an oncogene by regulation the BMI-1 pathway and c-Myc pathway

**Figure 5:**
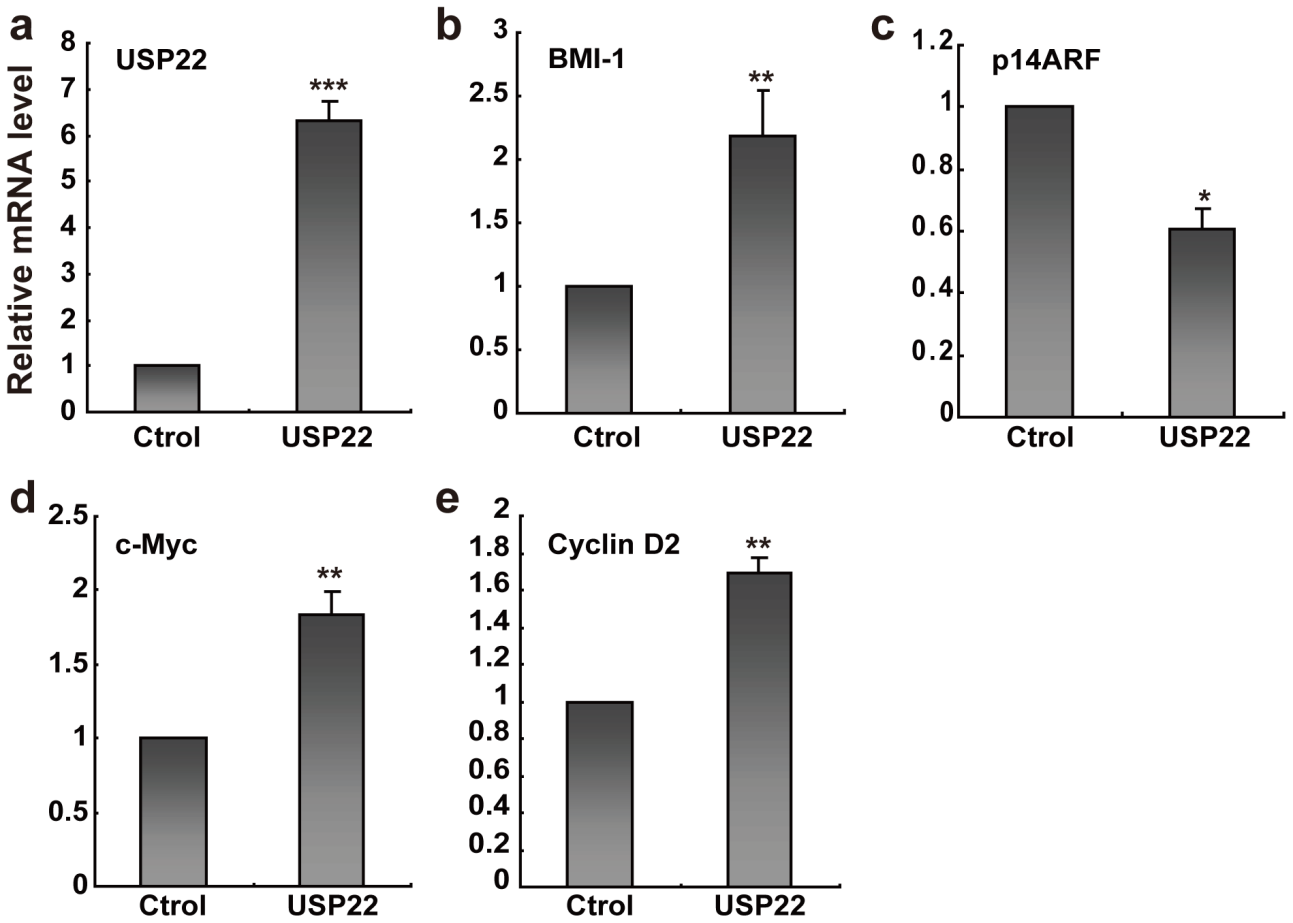
USP22 regulates the BMI-1 and c-Myc signaling pathway in ACC-83 cancer cells. Relative USP22, BMI-1, p14ARF, c-Myc and cyclin D2 mRNA expression in ACC-83 cells transiently transfected with Control vector (Ctrol) or Flag-USP22 vector (USP22) for 48 h. β-actin mRNA levels were used as an internal normalization control. *P<0.05, **P<0.01, ***P<0.001 using Student’s t test. Data are mean ± s.e.m. of triplicates from three independent experiments.

USP22 plays a central role in the control of cell cycle progression, gene transcription and tumorigenesis. We examined the transcriptional relationship in the BMI-1, p14ARF, c-Myc and cyclin D2 expression by quantitative real-time PCR in the ACC-83 cancer cells (Figure 5). Given the role of USP22 in the regulation of BMI-1 activity, we investigated the possibility that USP22 might be a relevant factor in SACC. First, upregulation of USP22 (Figure 5a) in the ACC-83 cell line resulted in the increase expression of BMI-1 (Figure 5b) and an overall downregulation of the BMI-1 target gene p14ARF (Figure 5c). To further investigate how the endogenous mRNA level of c-Myc/cyclin D2 pathway was affected by USP22, we performed real-time PCR experiments. The results showed that USP22 was able to promote an increase in the endogenous concentration of c-Myc and cyclin D2 mRNA (Figure 5d-e). This novel finding suggests that USP22 promotes cell proliferation via the c-Myc/cyclin D2 pathway, leading to cancer development.

## Discussion

To date, many studies demonstrate that USP22 represent a novel prognostic biomarker in tumor progression and oncogenesis [15]. USP22 is highly expressed in cancer tissues and is significantly associated with progression and unfavorable clinical outcome [16]. USP22, as an important member of histone deubiquitinating enzymes, is reported to remove ubiquitin moieties from histones H2A and H2B and regulates histone acetylation (HAT) complex SAGA [17]. Moreover, many reports suggested that USP22 is associated with cell-cycle progression via regulating INK4a/ARF pathway and Akt signaling pathway [12]. Xu et al. have revealed tha knockdown of USP22 by micro-RNA interference inhibits colorectal cancer growth [18]. Recent studies showed that increase expression of USP22 in salivary duct carcinoma patients were associated with a poor prognosis [8]. In the present study, we showed that the rate of high USP22 expression was significantly higher in SACC group than adjacent non-cancerous tissue.

SACC is a relatively commonly seen salivary gland tumor with high invasive nature, readily nerve and vascular involvement and high metastatic rate through circulation [19]. Clinical studies have established a variety of markers in SACC that predict poor prognosis, including advanced stage at diagnosis, the status of regional lymph node metastasis, positive surgical margin, and perineural invasion [20], [21]. However, these markers are based solely upon clinicopathological parameters and do not provide insight into the pathophysiological mechanisms guiding SACC growth and spread. Our study aimed to define USP22 expressed on SACC which have prognostic impact, and might be functionally involved in SACC progression and development.

In the present study, the expression and clinical significance of USP22 were first evaluated in 135 cases of SACC. The results showed that USP22 expression was correlated with histological subtype, lymph node metastasis, grade, Ki-67 and SOX2 expression, and the status of USP22 expression could be predictive factors of SACC. Interestingly, upregulation of USP22 accompanies with the regulation of the BMI-1 pathway and c-Myc pathway in ACC-83 cancer cell lines. Furthermore, our results show that high USP22 expression had low OS and DFS in patients with SACC. Multivariate analysis declared that USP22 was an independent prognostic factor for the OS and DFS. These findings suggest that high expression of USP22 might be able to predict a worse prognosis in SACC patients. In addition, the suppression of USP22 expression may provide a potential target for therapeutic intervention in SACC.

In conclusion, our study provides evidence that USP22 expression is associated with histological subtype, lymph node metastasis, grade, Ki-67 and SOX2 expression in SACC patients, and also is an independent prognostic factor for SACC. Nevertheless, the potential molecular mechanism between increased USP22 expression and cancer metastasis in SACC is still unknown. Further studies and more samples will be required to investigate the prognostic role of USP22 in SACC. Based on these data, we propose that USP22 may be an attractive and promising therapeutic target for SACC patients.
